# An Innovative Image Encryption Algorithm Based on the DNAS_box and Hyperchaos

**DOI:** 10.3390/e27030239

**Published:** 2025-02-26

**Authors:** Da Qiu, Tingting Zhang, Jingyi Liu, Song Liu, Peiyu He

**Affiliations:** 1 College of Electronics and Information Engineering, Sichuan University, Chengdu 610065, China; qiuda2008@163.com (D.Q.); liumole@163.com (J.L.); 2 School of Intelligent Systems Science and Engineering, Hubei Minzu University, Enshi 445000, China; 2014061@hbmzu.edu.cn (T.Z.); 2002016@hbmzu.edu.cn (S.L.)

**Keywords:** S_BOX, DNA coding, image encryption, chaos sequence

## Abstract

This study proposes an innovative image encryption algorithm based on the DNAS_box and hyperchaos. The algorithm dynamically constructs a DNAS_box using 2-bit deoxyribonucleic acid (DNA) coding and 4-bit DNA-like coding, enabling seamless conversion between these two coding schemes. The implementation process comprises four key phases. First, a hyperchaotic system generates chaotic sequences while dynamically encoding the plaintext image according to DNA coding rules. Next, the 2-bit DNA keys undergo encoding before performing XOR operations with the encoded plaintext image. Subsequently, under chaotic sequence control, the DNAS_box transforms 2-bit DNA symbols into 4-bit DNA-like symbols. Finally, XOR operations are executed between the 4-bit DNA-like symbols and corresponding DNA-like keys, producing a 4-bit DNA-like symbol sequence. This sequence is dynamically decoded under chaotic sequence guidance to generate the ciphertext image. The algorithm’s effectiveness is validated through MATLAB-based numerical simulations, with experimental results confirming its robust security performance. Notably, the algorithm demonstrates a key space of approximately 10^145^, an NPCR exceeding 99.5%, and ciphertext entropy surpassing 7.997.

## 1. Introduction

In recent years, various image encryption schemes based on diverse theories and technologies have been developed to enhance the security of digital images during transmission and storage. Notable among these are methods based on chaos [1,2,3,4], DNA coding [5,6,7,8], coupled mapping [9,10], S_box [11], and compressed sensing [12].

Recently, DNA technology has been commonly used in digital image encryption because of its advantages of large-scale parallelism, high information density, low power consumption, and no rounding error in image encryption based on DNA operation. In [13,14], an image encryption algorithm was developed by combining logistic chaotic mapping and DNA computing. In [15,16], image encryption schemes based on the DNA theory and double random phase coding (DRPE) technology were designed using different chaotic systems. In [17], an image encryption scheme based on a nested chaotic map and DNA was proposed. In [18], based on the traditional binary XOR operation, an advanced DNA sequence XOR operation was defined and combined with a one-way coupling mapping lattice (OCML) to develop an innovative, robust, and lossless color image encryption algorithm. The DNA coding and DNA operations denote the core of the aforementioned algorithms, which mainly include two parts: (1) employ the DNA coding theory to encode plaintext images and keys into DNA symbol sequences; and (2) subject two DNA symbol sequences to different operations (e.g., +, −, and XOR operations) to obtain a ciphertext image. The encryption algorithms based on DNA operations belong to stream ciphers, and there is a one-to-one relationship between the plaintext and ciphertext images. However, attackers can use this relationship to attack the encryption system [19,20,21]. Liu et al. [22] used only part of the known plaintext or ciphertext to reconstruct an equivalent key and cracked an encryption system using DNA technology. Hermassi et al. [21] pointed out that the image encryption algorithm based on DNA coding and two chaotic maps proposed in [23] has serious defects; that is, this encryption method is irreversible and cannot resist known plaintext attacks. In ref. [24], an image fusion encryption scheme based on the Chen hyperchaos and DNA sequences was proposed. However, because the keystream was generated independently of plaintext, it was deciphered by the selected plaintext attack in [19,25].

In ref. [20], the authors studied the security problem in symmetric encryption algorithms based on DNA coding, and it was concluded that the encryption algorithm based on the DNA encoding–decoding model had only eight equivalent key pairs. In addition, due to the small key space, it was difficult for this type of algorithm to resist brute force attacks and known plaintext attacks. The encryption algorithms based on a stream cipher model have a one-to-one correspondence between the plaintext and ciphertext, which makes it difficult to resist selected plaintext attacks. However, the combination of DNA technology and chaos theory can effectively enhance security. In ref. [26], it was pointed out that if a fixed DNA encoding rule is employed for encryption, the distribution of plaintext bits cannot change. This phenomenon is even more obvious when processing medical images. When attackers use special image attacks, the anti-attack ability is relatively weak, especially for monochrome images and all-black or all-white images. Therefore, some encryption schemes adopt dynamic selection of DNA coding rules in the DNA encoding and decoding processes [27,28,29]. In ref. [30], a DNA-like coding scheme that uses a 4-bit binary sequence to represent 16 characters was proposed; the number of effective coding rules was expanded to 1024, but the encoding–decoding model was still used in the DNA-like diffusion process of this scheme. In ref. [29], the authors pointed out that image encryption algorithms based on DNA encoding usually have security defects, which can be summarized as follows: (1) an equivalent key can be obtained through a pair of plaintext images and the corresponding ciphertext images; (2) the encryption process is not sensitive to changes in plaintext images or keys; and (3) the employed rules of DNA encoding and decoding in the encryption process are fixed.

Aiming to overcome the above shortcomings of the encryption method based on DNA, this paper proposes a dynamic image encryption algorithm based on the DNAS-box and hyperchaos. The main features of the proposed algorithm are as follows. First, DNA coding and DNA-like coding are used to construct a DNAS_box, which can realize the conversion of 2-bit DNA symbols to 4-bit DNA-like symbols during the encryption process. Second, in the DNAS_box, the encoding and decoding rules in the encryption process are effectively separated, and the number of encoding and decoding rules is increased. Third, to improve the plaintext sensitivity of the proposed algorithm, the keys are closely related to the SHA-256 hash values of a plaintext image. Finally, in the encryption process, the encoding and decoding of DNA and DNA-like and the DNAS_box construction are dynamically controlled by chaotic sequences.

The rest of this paper is organized as follows. In Section 2, DNA coding, DNA-like coding, and the DNAS_box are described. In Section 3 and Section 4, the hyperchaos system used in this study and its initial value and parameter generation process are described. The detailed process of the proposed encryption algorithm is introduced in Section 5. The results of simulation experiments and security analyses are presented in Section 6 and Section 7. Finally, the main conclusions of this paper are presented in Section 8.

## 2. DNAS_box

### 2.1. DNA Coding

DNA is a long polymer composed of repeating nucleotide units that include A (adenine), G (guanine), C (cytosine), and T (thymine). According to the complementary principle of DNA, A and T, as well as G and C, are complementary to each other. In a computer, information is stored in a binary form, using only two basic symbols, “0” and “1”, which denote complementary relations. In 2-bit encoding, 00 and 11, as well as 10 and 01, represent complementary relations. A DNA nucleobase (A C G T) can form a one-to-one mapping with 2-bit coding (00 01 10 11). There are 4! = 24 types of maps, but only 8 types of maps can satisfy the complementary relationship [20], as shown in Table 1.

Assume an encoding–decoding encryption method is applied to an 8-bit gray image with a pixel gray value of 180, which corresponds to a binary sequence “10110100”. According to Rule5 in Table 1, this image can be converted into a DNA sequence, denoted by TGAC. Then, by using Rule 3 for decoding, a new binary sequence, “00101101”, is obtained, which corresponds to the decimal number 45. If the plaintext image is encoded using Rule K1 and then decoded following Rule K2, thus hiding the original information. The security of this encryption method depends on Rules K1 and K2 because (K1, K2) pairs of equivalence exist, and there are only eight valid (K1, K2) pairs [20]. To improve the security of the implementation of DNA into image encryption algorithms, it is also possible to perform the XOR, addition, and subtraction operations on DNA sequences [6,7,8]. The XOR operation is used in the algorithm proposed in this study, as shown in Table 2.

### 2.2. DNA-like Coding

In ref. [30], a DNA-like coding method, which uses a 4-bit binary sequence to represent 16 characters, was proposed. This coding method uses 16 characters, namely A, B, C, D, E, F, G, H, I, J, K, L, M, N, O, and P characters, to represent DNA-like symbols, where A and B, C and D, E and F, G and H, I and J, K and L, M and N, and O and P are complementary. Therefore, there are 16 corresponding 4-bit binary sequences: 0000, 0001, 0010, 0011, 0100, 0101, 0110, 0111, 1000, 1001, 1010, 1011, 1100, 1101, 1110, and 1111. Further, the DNA-like symbols and 4-bit binary sequences have a one-to-one mapping relationship. In addition, according to the principle of complementarity, there will be 1024 coding schemes, which can significantly increase the difficulty of the attack on DNA coding. There are eight types of DNA-like coding schemes, as shown in Table 3. Similarly, DNA-like coding can still be performed using the XOR, addition, and subtraction operations. Taking Rule 1 in Table 3 as an example, Table 4 provides a DNA-like XOR operation rule.

### 2.3. Dynamic DNAS_box

A substitution box (S-box) is a very effective nonlinear replacement tool. In this study, a DNAS_box is constructed by combining DNA and DNA-like coding to achieve a dynamic conversion between these two types of coding. The encoding and decoding rules in an image encryption algorithm are effectively isolated to improve the security of the encryption algorithm. An example of a dynamic DNAS_box is presented in Table 5. The DNA coding rules in the S_box are dynamically selected from Table 1, whereas the DNA-like coding rules are dynamically selected from Table 3; the order of DNA-like symbols is dynamically controlled by chaotic sequences, which constitute the DNAS_box in Table 5.

**Figure 1:**
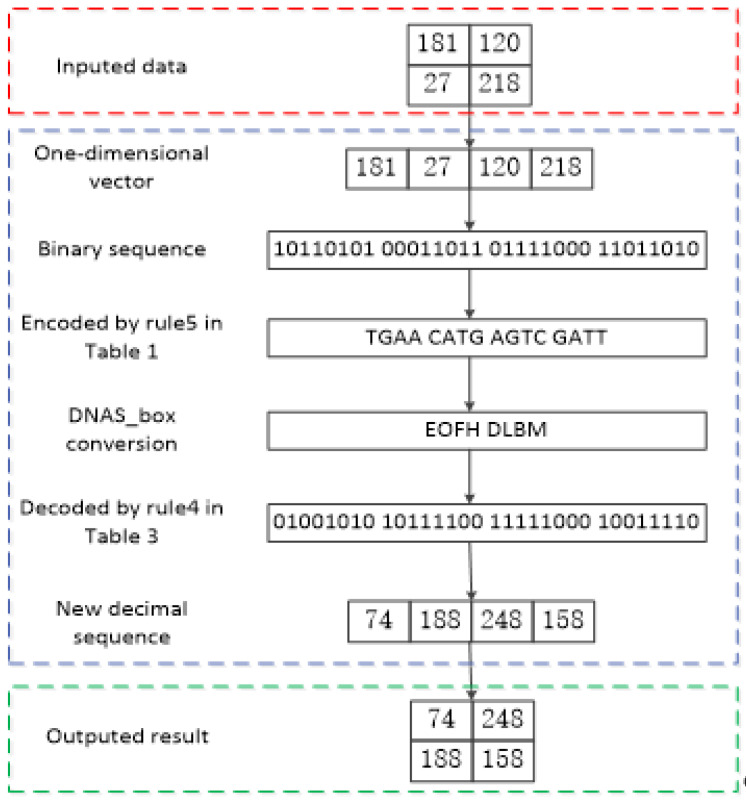
Example of DNAS_box encoding applied to image encryption.

For instance, assume that the pixel value of a plaintext image is 172, which can be converted into a binary sequence “10101100” and encoded by Rule 6 in Table 1 to obtain the DNA sequence AAGC. Then, the DNA sequence AAGC is divided into two groups, [AA] and [GC], which represent the row and column of DNA S_box, respectively; the DNA-like symbols corresponding to [AA] and [GC] are respectively denoted by L and J, as shown in Table 5. Afterward, the binary sequence “11101001” is obtained by decoding following Rule 2 in Table 3, which denotes the decimal number 233. Finally, the encryption process is complete. The process of DNAS_box conversion in the image encryption process is illustrated in Figure 1. The red dashed box contains the data to be encrypted, the blue dashed box details the DNAS_box encoding encryption process, and the green dashed box represents the encrypted data.

## 3. Chen Hyperchaos System

**Figure 2:**
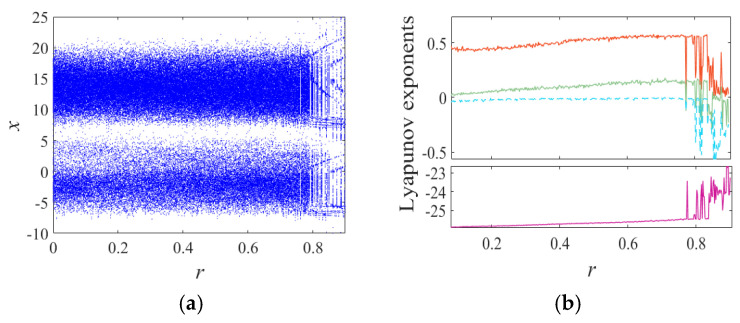
The bifurcation diagram and Lyapunov exponents diagram of system (1). (**a**) Bifurcation diagram for increasing *r* and (**b**) the Lyapunov exponents versus *r*.

**Figure 3:**
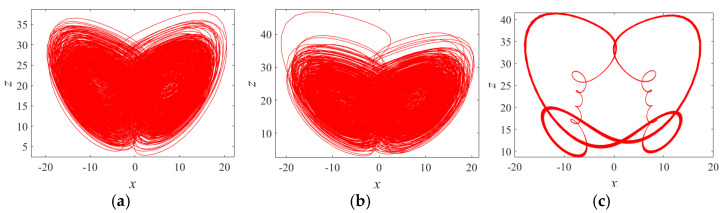
Phase portraits of system (1) with different *r*: (**a**) *r* = 0.03; (**b**) *r* = 0.58; (**c**) *r* = 0.87. (**a**) Chaotic attractor, (**b**) hyperchaotic attractor, and (**c**) period orbit.

The dynamic behavior of a high-dimensional hyperchaos system is complex, and an image encryption algorithm designed using this system has an improved security effect. This study employs the Chen hyperchaos system [31], which can be expressed as follows:

(1) $$\left\{\begin{array}{l} \dot{x}=a(y-x)+w\\ \dot{y}=dx-xz+cy\\ \dot{z}=xy-bz\\ \dot{w}=yz+rw \end{array}\right.$$

where *a, b, c, d*, and *r* are system control parameters, and *x, y, z*, and *w*, are system (1) state variables. The bifurcation diagram and Lyapunov exponent spectrum for the Chen system with parameters a=35, b=3, c=12, d=7, and initial values (0.2, 0.1, 0.1, 0.1) over the range 0<r≤0.9 are shown in Figure 2. When 0<r≤0.085 and 0.836<r≤0.854, the system exhibits one positive Lyapunov exponent, indicating a chaotic state. For 0.085<r≤0.836 and 0.874<r≤0.886, the system has two positive Lyapunov exponents, corresponding to a hyperchaotic state. In the intervals 0.854<r≤0.874 and 0.886<r≤0.9, the maximum Lyapunov exponent becomes zero, signifying a periodic state. To further illustrate the influence of parameter r, phase diagrams corresponding to r=0.03, 0.58, and 0.87 are provided in Figure 3.

## 4. SHA-256 and Initial Conditions

The secure hash algorithm SHA-256 represents a hash function, and it has been widely used in encryption due to its good security. It can generate 256-bit message digests based on the input image, which are usually represented by a 64-bit hexadecimal number sequence $H=(h_{1},h_{2},\cdots ,h_{64})$. Even if there is a slight change in a plaintext image, completely different message digests can be obtained. In this study, a message digest H is generated by the SHA-256 from a plaintext image, and the XOR operation is performed using an externally input 256-bit key $\mathrm{INK}=(ink_{1},ink_{2},\cdots ,ink_{64})$ to obtain $K=(k_{1},k_{2},\cdots ,k_{64})$. According to Equations (2) and (3), the initial values and parameters of chaos (1) iteration are generated as keys. Equations (2) and (3) are defined as follows:

(2) $$\left\{\begin{array}{c} a=\frac{\left(k_{1}⊕k_{2}⊕L⊕k_{8}\right)+\left(k_{9}⊕k_{10}⊕L⊕k_{16}\right)}{2^{5}+2}\;\\ b=\frac{\sum_{i=17}^{24}k_{i}⊕\sum_{i=25}^{32}k_{i}}{2^{8}+1}\;\\ \;c=\frac{\left(k_{33}⊕k_{34}⊕L,⊕k_{40}\right)+\left(k_{41}⊕k_{42}⊕L,⊕k_{48}\right)}{2^{5}+2}\;\\ d=\frac{\sum_{i=49}^{56}k_{i}⊕\sum_{i=57}^{64}k_{i}}{2^{8}+1}\; \end{array}\right.$$

and

(3) $$\left\{\begin{array}{c} x_{0}=inx+\frac{a+b+c}{3}\\ y_{0}=iny+\frac{a+b+d}{3}\\ z_{0}=inz+\frac{a+c+d}{3}\\ w_{0}=inw+\frac{b+c+d}{3}\\ \;r_{0}=inr+\frac{a+b+c+d}{4} \end{array}\right.$$

where ⊕ denotes the XOR operation; *inx, iny, inz,* and *inw* are the initial values of chaotic system (1) with the external input; inr is the parameter of chaotic system (1) with the external input; *x*_0_*, y*_0_*, z*_0_, and *w*_0_ are the initial values of chaotic system (1) during the iteration process; and *r*_0_ is the parameter of chaotic system (1) during the iteration process.

## 5. Encryption and Decryption Processes

**Figure 4:**
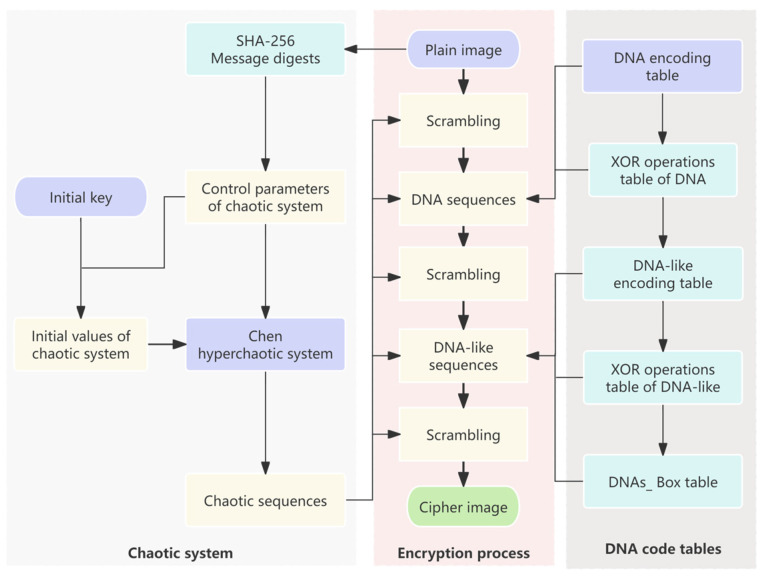
The scheme for image encryption.

**Figure 5:**
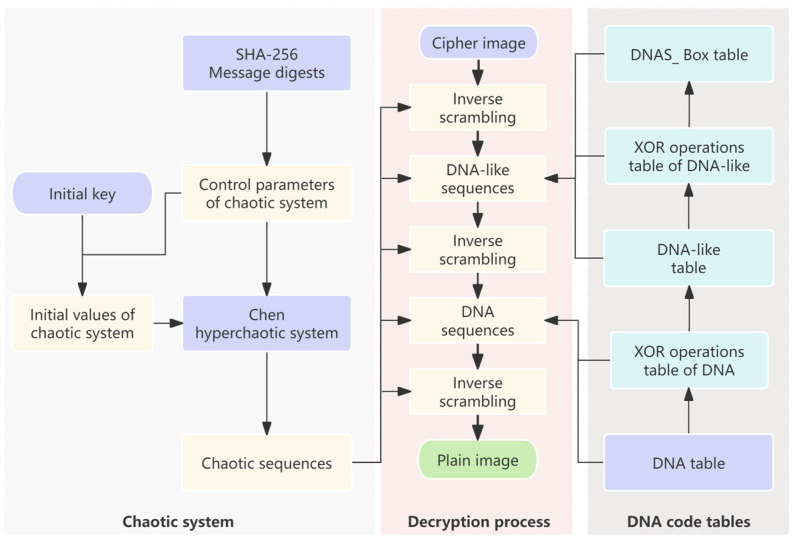
The scheme for image decryption.

The proposed encryption scheme is given in Figure 4. The Chen hyperchaos system is used to generate random sequences, and DNA encoding and DNAS_box construction are dynamically controlled, as shown in steps 1–20. Decryption represents the reverse operation of encryption, and the decryption process of the proposed algorithm mainly includes three parts: DNA-like decoding and reverse operation, DNAS_box reverse operation, and DNA decoding and reverse operation. The proposed decryption scheme is shown in Figure 5. The encryption system employs a symmetric cryptographic scheme, where the message digest and the initial key needs to be transmitted to the receiver via a secure channel.

Step 1: Input a plaintext image **P** with an image size of m×n;

Step 2: The message digests of image P are extracted by SHA-256, and a 64-bit hexa decimal number sequence H is obtained; then, H is subjected to the XOR operation with the external input key INK, and the parameters and initial values of chaotic system (1) during iteration are determined by Equations (2) and (3);

Step 3: Chaotic system (1) is iterated (l+8×m×n) times. In addition, to eliminate the transient effect of the chaotic system, the first l terms are discarded, and four chaotic sequences x, y, z and w, with a length of 8×m×n, are obtained;

Step 4: Plaintext image **P** is converted into a one-dimensional row vector **P_1_**;

Step 5: Four subsequences x_1_, x_2_, x_3_ and x_4_ of chaotic sequence x, having the length of m×n, are obtained by using Equation (4);

(4) $$\left\{\begin{array}{ll} x_{1}\left(i\right)=x\left(\left(i-1\right)\times 8+2\right) & \\ x_{2}\left(i\right)=x\left(\left(i-1\right)\times 8+4\right) & \\ x_{3}\left(i\right)=x\left(\left(i-1\right)\times 8+6\right) & \\ x_{4}\left(i\right)=x\left(\left(i-1\right)\times 8+8\right) & \end{array}\right.\;i=1,2,\ldots ,m\times n$$

Step 6: Chaotic sequence x_1_ is sorted from small to large, forming a position sequence s_1_. The pixel position of **P_1_** is scrambled using Equation (5), and a one-dimensional vector **P_2_** is obtained;

(5) $$P_{2}\left(i\right)=P_{1}\left(s_{1}\left(i\right)\right)$$

Step 7: Vector P_2_ is decomposed into a binary 0–1 symbol sequence **P_3_**, and the chaotic sequence x is sorted in ascending order to obtain a position sequence s_2_; **P_3_**, is subjected to the position scrambling operation using the method in Step 6 to obtain a binary sequence;

Step 8: By using Equation (6), the integer composed of 9–12 digits after the decimal point of x_2_ is extracted, and the modulo operation is performed on 256 to obtain a 0–255 integer sequence kx_2_. Then, kx_2_ is decomposed into a binary sequence kx_21_.

(6) $${kx}_{21}\left(i\right)=mod\left(floor\left(x_{2}\times 10^{12}\right)-floor\left(x_{2}\times 10^{8}\right)\times 10000,256\right)$$

where floor(x) is the largest integer less than x, and mod(x,y) is the remainder of x divided by y;

Step 9: Two subsequences of a chaotic sequence y, denoted by y_1_ and y_2_, are obtained using Equation (7), and they both have a length of 4×m×n; ky_1_ and ky_2_ are two 1–8 random number sequences obtained using Equation (8) from y_1_ and y_2_, respectively; ky_1_ and ky_2_ are used to dynamically select the encoding rules from Table 1, when the binary sequences $P_{4}$ and kx_21_ are used to perform DNA encoding, respectively;

(7) $$\left\{\begin{array}{ll} y_{1}\left(i\right)=y\left(2\times i-1\right) & \\ y_{2}\left(i\right)=y\left(2\times i\right) & \end{array}\right.\;i=1,2,\ldots 4\times m\times n$$

(8) $$\left\{\begin{array}{ll} {ky}_{1}=mod\left(floor\left(y_{1}\times 10^{12}\right)-floor\left(y_{1}\times 10^{10}\right)\times 100,8\right)+1 & \\ {ky}_{2}=mod\left(floor\left(y_{2}\times 10^{12}\right)-floor\left(y_{2}\times 10^{10}\right)\times 100,8\right)+1 & \end{array}\right.$$

Step 10: After performing dynamic DNA encoding on $P_{4}$ and kx_21_, two DNA symbol sequences $P_{5}$ and kx_22_ are obtained. The XOR operation is carried out on $P_{5}$ and kx_22_ following the rules in Table 3, and the DNA symbol sequence $P_{6}$ is obtained;

Step 11: A position sequence s_3_ is obtained by sorting y_1_ in ascending order, and a DNA symbol sequence $P_{7}$ is obtained by scrambling $P_{6}$ using the method of Step 6;

Step 12: A subsequence z_1_ is extracted from a chaotic sequence z using Equation (9); it has a length of 16 and is sorted in ascending order to obtain a position sequence s_4_. According to the method of Step 6, the position of the DNA-like coding symbol {ABCDEFGHIJKLMNOP} is scrambled and transformed into a 4×4 matrix. In this way, the DNAS_ box is obtained, as shown in Table 5;

(9) $$z_{1}\left(i\right)=z\left(i\times 32+2\right)\;i=1,2,\ldots ,16$$

Step 13: $P_{7}$ is divided into two subsequences $P_{71}$ and $P_{72}$, with a length of 2×m×n; $P_{71}$ and $P_{72}$ are used as row and coordinates, respectively. The corresponding DNA-like coding symbols are presented in Table 5, and a DNA-like coding symbol sequence, $P_{8}$ is composed;

Step 14: By using the method of Step 8, an integer composed of 9–12 digits after the decimal point of x_3_ is extracted; also, the modular operation is performed on 256, and a 0–255 integer sequence kx_3_ is obtained, which is then decomposed into a binary sequence kx_31_;

Step 15: A chaotic sequence y is sorted in ascending order, and a position sequence s_5_ is obtained. The position of kx_31_ is adjusted using the method of Step 6, and a new binary sequence kx_32_ is obtained;

Step 16: Two sequences kz_1_ and kz_2_ are extracted from a chaotic sequence z using Equation (10), and they both have a length of 2×m×n. The two sequences are converted into 1–8 random number sequences kz_1_ and kz_2_ using Equation (11); then, kz_1_ is used to dynamically select the coding rules from Table 3, kx_32_ is encoded into a DNA-like sequence, and kz_2_ is used to dynamically select the decoding rules from Table 3 when the DNA-like sequences are decoded in Step 18;

(10) $$\left\{\begin{array}{ll} {{kz}_{1}}^{\prime }\left(i\right)=z\left(\left(i-1\right)\times 4+1\right) & \\ {{kz}_{2}}^{\prime }\left(i\right)=z\left(\left(i-1\right)\times 4+3\right) & \end{array}\right.\;i=1,2,\ldots ,2\times m\times n$$

(11) $$\left\{\begin{array}{ll} {kz}_{1}=mod\left(floor\left({kz}_{1}^{\prime }\times 10^{12}\right)-floor\left({kz}_{1}^{\prime }\times 10^{10}\right)\times 100,8\right)+1 & \\ {kz}_{2}=mod\left(floor\left({kz}_{2}^{\prime }\times 10^{12}\right)-floor\left({kz}_{2}^{\prime }\times 10^{10}\right)\times 100,8\right)+1 & \end{array}\right.$$

Step 17: Afterward, kx_32_ is dynamically encoded by DNA-like coding, and a DNA-like coding symbol sequence kx_33_ is obtained. Further, the DNA-like XOR operation is performed on $P_{8}$ and kx_33_ following the rules in Table 4, and a sequence $P_{9}$ is obtained;

Step 18: Sequence $P_{9}$ is decoded following the DNA-like decoding rules dynamically selected from Table 3 based on kz_2_, and a binary sequence $P_{10}$ is obtained;

Step 19: A chaotic sequence w is sorted in ascending order, and a position sequence s_6_ is obtained. According to the method of Step 6, the position of $P_{10}$ is scrambled, and a binary sequence $P_{11}$ is obtained;

Step 20: Next, the binary sequence $P_{11}$ is transformed into an 8-bit decimal sequence $P_{12}$; chaotic sequence x_4_ is sorted in ascending order to obtain a position sequence s_7_. Using the method of Step 6, $P_{12}$ is scrambled to obtain a decimal sequence $P_{13}$, which is then transformed into a two-dimensional image matrix, with a size of m×n. Then, the final ciphertext image is obtained.

## 6. Simulation Experiments

**Figure 6:**
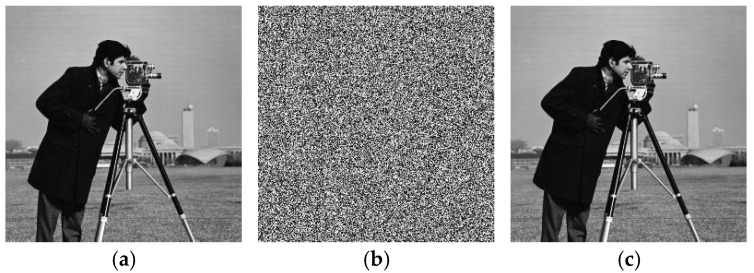

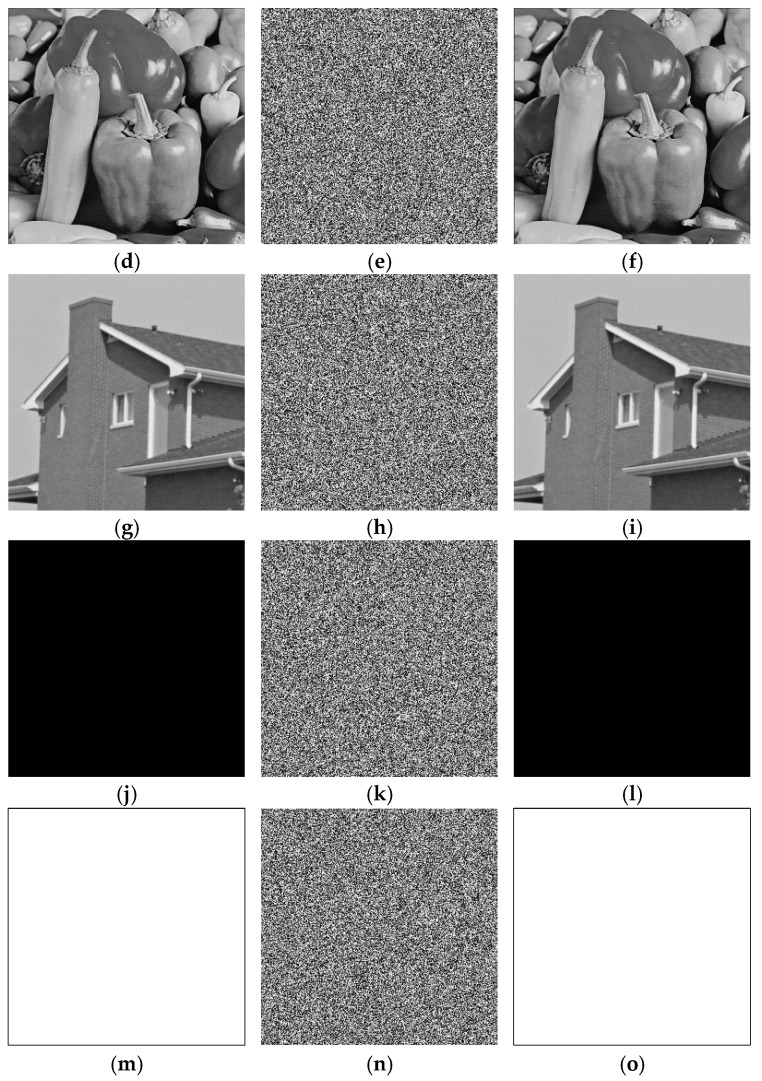
Simulation experiment results. (**a**) Cameraman plaintext image, (**b**) Cameraman ciphertext image, (**c**) Cameraman decrypted image, (**d**) Peppers plaintext image, (**e**) Peppers ciphertext image, (**f**) Peppers decrypted image, (**g**) House plaintext image, (**h**) House ciphertext image, (**i**) House decrypted image, (**j**) all-black plaintext image, (**k**) all-black ciphertext image, (**l**) all-black decrypted image, (**m**) all-white plaintext image, (**n**) all-white ciphertext image, and (**o**) all-white decrypted image.

The feasibility of the proposed algorithm was verified by simulation experiments using classical images, namely Cameraman, Peppers, House, and all-black and all-white images, with a size of 256×256. The experimental platform was as follows. The hardware part included an AMD PRO A12-9800 R7 3.80 GHz CPU (Advanced Micro Devices, Inc., Santa Clara, CA, USA) and an 8 GB DDR4 RAM (Kingston Technology Corporation, Fountain Valley, CA, USA).; the software part included the Windows 10 operating system and MATLAB R2016A software. The experimental results are shown in Figure 6, where it can be seen that the ciphertext images were in a noisy state, without any plaintext image information displayed, and the decrypted images were visually indistinguishable. After the numerical analysis, the difference between the plaintext image and the ciphertext image was zero.

## 7. Security Analysis

### 7.1. Key Space Analysis

An encryption algorithm should have enough key space to resist brute force attacks, which usually should be larger than 2^100^ [27,32].

The main parameters of the proposed algorithm include the following:

(1) The initial values inx, iny, inz, inw, and a parameter inr of the externally input chaotic system;

(2) An externally input 256-bit key INK;

(3) The 256-bit hash values of the plaintext image generated by SHA-256.

The accuracy of the initial values inx, iny, inz, inw, and parameter inr of the chaotic system was calculated as 10^14^, and the key space’s size was 10^70^. In addition, the key space of the plaintext image generated by SHA-256 was 2^128^, the external key INK was 256 bits, and the key space was 2^128^. Thus, the total key space was S = 2^128^ × 2^128^ × 10^70^ ≈ 10^145^ > 2^100^, indicating that the proposed algorithm had a large enough key space to resist brute force attacks.

### 7.2. Key Sensitivity Analysis

The key sensitivity includes encryption sensitivity and decryption sensitivity. In the encryption process, the key sensitivity is reflected by the Number of Pixels Change Rate (NPCR) and the unified average changing intensity (UACI) between the ciphertext image obtained after minor changes in the key and the original ciphertext image. To test the encryption key sensitivity of the proposed algorithm, the Cameraman image was used, and the external key was made by making slight changes (increase $1\times 10^{-14}$) during encryption; then, the NPCR and UACI were calculated between the new ciphertext image and the original ciphertext image using Equation (12). The results are shown in Table 6.

(12) $$\begin{array}{r} NPCR=\frac{\sum_{i,j}^{M,N}D\left(i,j\right)}{M\times N}\times 100\%,\;D\left(i,j\right)=\left\{\begin{array}{ll} 1,C_{1}\left(i,j\right)\neq C_{2}\left(i,j\right) & \\ 0,C_{1}\left(i,j\right)=C_{2}\left(i,j\right) & \end{array}\right.\\ UACI=\frac{\sum_{i,j}^{M,N}\left|C_{1}\left(i,j\right)-C_{2}\left(i,j\right)\right|}{M\times N\times 255}\times 100\% \end{array}$$

where M and N represent the number of image rows and columns, respectively; and C_1_ and C_2_ are two ciphertext images encrypted with a key difference of $1\times 10^{-14}$.

The results in Table 6 show that when the key changed slightly, the NPCR reached more than 99.5%, and the UACI was larger than 33.4%, which indicated that the proposed algorithm had good key sensitivity in the encryption process.

.

**Figure 7:**
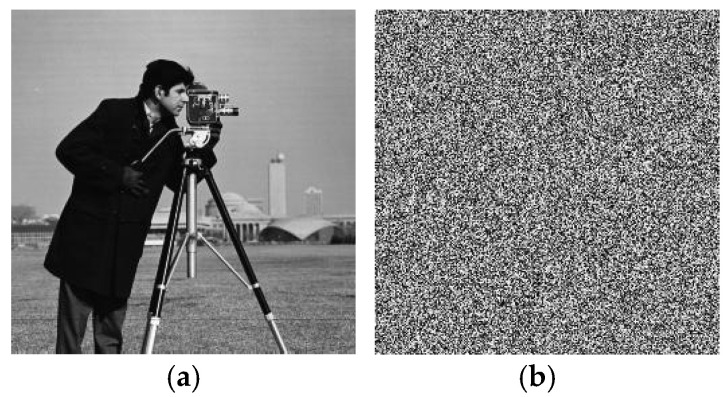
Decryption key sensitivity test. (**a**) Image decrypted with key A and (**b**) image decrypted with key B.

In the decryption process, even when the key changed slightly, the plaintext image information could not be restored. The result of using external key B (x_0_ = 1.256, y_0_ = 1.387, z_0_ = 1.566, w_0_ = 1.867, r_0_ = 0.16 + 10^−14^) to restore the image encrypted with key A (x_0_ = 1.256, y_0_ = 1.387, z_0_ = 1.566, w_0_ = 1.867, r_0_ = 0.16) is shown in Figure 7, where it can be seen that effective plaintext image information could not be obtained even if the key changed only slightly; thus, the proposed algorithm had good key sensitivity in the decryption process.

### 7.3. Histogram Analysis

**Figure 8:**
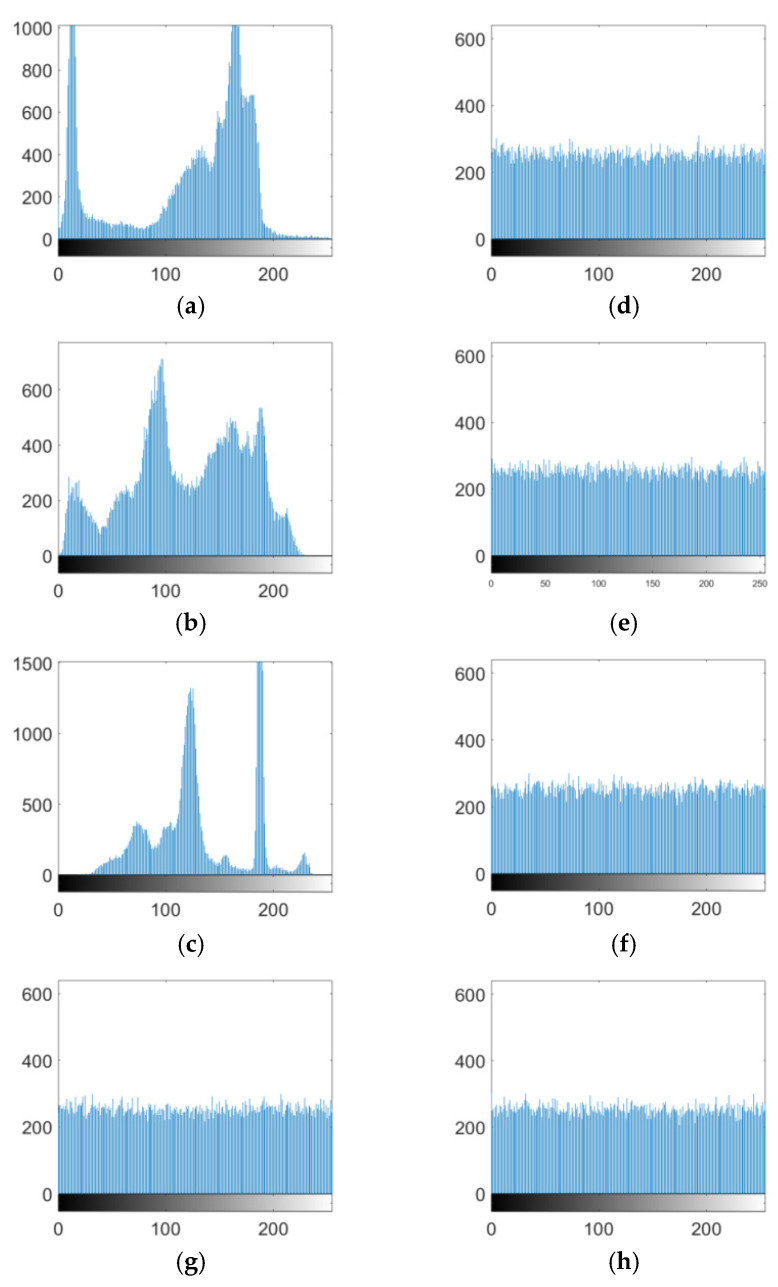
Histogram of the images. (**a**) Cameraman plaintext histogram. (**b**) Peppers plaintext histogram. (**c**) House plaintext histogram. (**d**) Cameraman ciphertext histogram. (**e**) Peppers ciphertext histogram. (**f**) House ciphertext histogram. (**g**) all-black ciphertext histogram. (**h**) all-white ciphertext histogram.

The histogram of an image intuitively shows the distribution of image pixel values. If the histogram is more evenly distributed, the statistical features of an image are smaller, and the corresponding encryption algorithm is more resistant to statistical attacks and vice versa. In this study, Cameraman, Peppers, House, all-black images, and all-white images were encrypted. The histograms of the plain text images Cameraman, Peppers, and House are shown in Figure 8a–c, and the histograms of the ciphertext images Cameraman, Peppers, House, all-black image, and all-white image are shown in Figure 8d–h, where it can be seen that the histogram of the ciphertext image was very uniform, which commendably hid the statistical information characteristics on the image.

### 7.4. Information Entropy Analysis

Information entropy is a measure of the randomness of information, which can be used to analyze the uncertainty of information in an encrypted image quantitatively. For an 8-bit grayscale image P as an information source, information entropy is calculated as follows:

(13) $$H\left(P\right)=-\sum^{255}p\left(x_{i}\right){log}_{2}p\left(x_{i}\right)$$

where H(P) represents the information entropy of grayscale image P, and $P(x_{i})$ is the probability of image pixel value x_i_.

For an 8-bit grayscale image, the ideal value of information entropy is eight [33,34]. The closer the entropy is to the value of eight, the more random image information is distributed, and the less likely information leakage is. The values of information entropy of ciphertext images obtained by the proposed algorithm and the comparison results with the methods proposed in [6,7,27,35,36] are presented in Table 7, where the values of [27,35] denote the experimental results given in [7]. Based on the results in Table 7, the information entropy of the ciphertext images obtained by the proposed algorithm was closer to the ideal value of eight than those of the other methods, which indicated that the image information encrypted by the algorithm had strong randomness.

### 7.5. Adjacent Pixels Correlation

**Figure 9:**
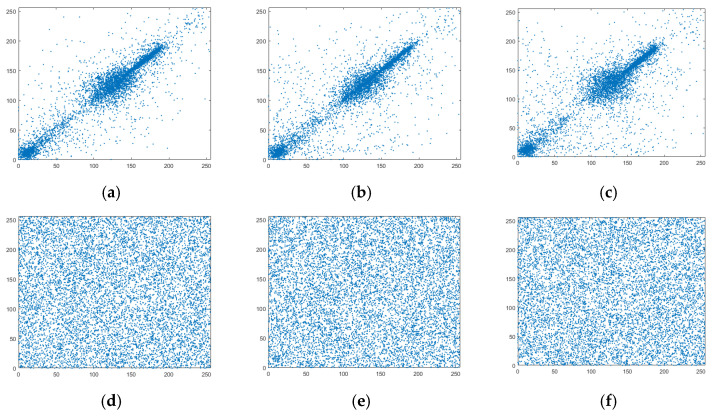
Correlation comparison of adjacent pixels in Cameraman. (**a**) plaintext horizontal, (**b**) plaintext vertical, (**c**) plaintext diagonal, (**d**) ciphertext horizontal, (**e**) ciphertext vertical, and (**f**) ciphertext diagonal.

The adjacent pixels of a digital image have a strong correlation. Therefore, to resist a statistical attack, it is necessary to break this strong correlation. In this study, 10,000 pairs of adjacent pixels were randomly selected in the horizontal, vertical, and diagonal directions from plaintext images and images, and the correlation coefficients of the plaintext and ciphertext of five images were calculated as follows:

(14) $$\begin{array}{l} E\left(x\right)=\frac{1}{N}\sum_{i=1}^{N}x_{i}\\ D\left(x\right)=\frac{1}{N}\sum_{i=1}^{N}{\left(x_{i}-E\left(x\right)\right)}^{2}\\ Cov\left(x,y\right)=\frac{1}{N}\sum_{i=1}^{K}\left(x_{i}-E\left(x\right)\right)\left(y_{i}-E\left(y\right)\right)\\ r_{xy}=\frac{Cov\left(x,y\right)}{\sqrt{D\left(x\right)}*\sqrt{D\left(y\right)}} \end{array}$$

where *x* and *y* denote the gray values of two adjacent pixels of an image, N is the total number of pixel pairs selected from an image, and E(*x*) and D(*x*) represent the mean and variance of a vector *x*, respectively. The calculation results are shown in Table 8, where it can be observed that the correlation coefficients between the adjacent pixels in the horizontal, vertical, and diagonal directions of the plaintext image were all close to one, indicating a strong correlation. However, the correlation coefficients of the ciphertext image were less than 0.01, which was very close to zero; this indicated that the strong correlation between the adjacent pixels in this image was broken by the encryption algorithm, and the attacker could not acquire useful information from the ciphertext image through a statistical attack. A comparison of the correlation between the plaintext and the ciphertext of the Cameraman image is presented in Figure 9, where it can be seen that the points in the three directions of the plaintext image were concentrated near the diagonal. This indicated that the adjacent pixels were very similar and had a strong correlation. The points in the three directions of the ciphertext image were evenly covered in the whole space, which demonstrated that the strong correlation between the adjacent pixels of the image encrypted by the proposed algorithm was broken, effectively hiding the statistical information on the image.

### 7.6. Plaintext Sensitivity Analysis

Plaintext sensitivity is a performance index that reflects the difference between two slightly different plaintext images that are encrypted with the same key. It is crucial for resisting differential attacks and selective plaintext attacks, and it is generally determined using the NPCR and UACI values. It should be noted that for 256-bit grayscale images, the ideal values of NPCR and UACI are 99.6094% and 33.4635%, respectively [37].

To test the plaintext sensitivity of the proposed algorithm, this study selected the Cameraman, Peppers, and House images as test images. Each time, a pixel was randomly selected from the image. The pixel value was first added or subtracted by one and then encrypted with the same key. Next, the NPCR and UACI values of the ciphertext image and the original ciphertext image were calculated. Each image was tested 20 times, and the maximum, minimum, and average values of the test results were obtained, as shown in Table 9, where a comparison with the existing literature is provided. Based on the results in Table 9, the difference between the maximum and minimum values of the NPCR was within 0.15%, which indicated that the encryption performance of the proposed algorithm was very stable. The average value of the NPCR was very close to the ideal value, which verified that the proposed algorithm had a good ability to resist differential and selective plaintext attacks.

Attackers always use special images, such as all-white, all-black, or monochrome images, to attack an encryption method, trying to obtain some useful information [33]. In this study, 256 × 256 all-white and all-black images were used as test images. The histogram of the ciphertext image is shown in Figure 8. The entropy and correlation coefficient of the adjacent pixels are presented in Table 7 and Table 8, respectively, where it can be seen that the ciphertext image had a noisy state, which differed completely from the plaintext image. In addition, its histogram distribution was random, and no useful information could be obtained from the analysis of the ciphertext image. Moreover, the entropy of the ciphertext image was larger than 7.997, and the correlation coefficient of the three directions was close to zero. All these showed that the proposed algorithm has a good encryption effect on all-white and all-black images, ensuring high security.

### 7.7. Noise Attack

During data transmission, noise pollution is often encountered. Compared with other noises, salt and pepper noise has a greater impact on ciphertext images [7]. To test the anti-noise ability of the proposed algorithm, this study added the salt and pepper noise with a noise intensity of 0.005, 0.01, 0.05, 0.1, 0.2, and 0.3 to ciphertext images, in turn, and then decrypted the images. The peak signal-to-noise ratio (PSNR) was selected for quantitative evaluation, and it was calculated as follows:

(15) $$MSE=\frac{1}{m\times n}\sum_{i=1}^{m}\sum_{j=1}^{n}{\left[P\left(i,j\right)-D\left(i,j\right)\right]}^{2}$$

(16) $$PSNR=10\times lg\left(\frac{255^{2}}{MSE}\right)$$

where P is the original image, D is the decrypted image, and m and n denote the number of rows and columns of the image, respectively.

The experimental results are shown in Table 10, where it can be seen that when the intensity of the salt and pepper noise reached 0.3, the decrypted image could still recognize part of the information visually, indicating that the proposed algorithm had a strong ability to resist salt and pepper noise attacks.

### 7.8. Clipping Attack

**Figure 10:**
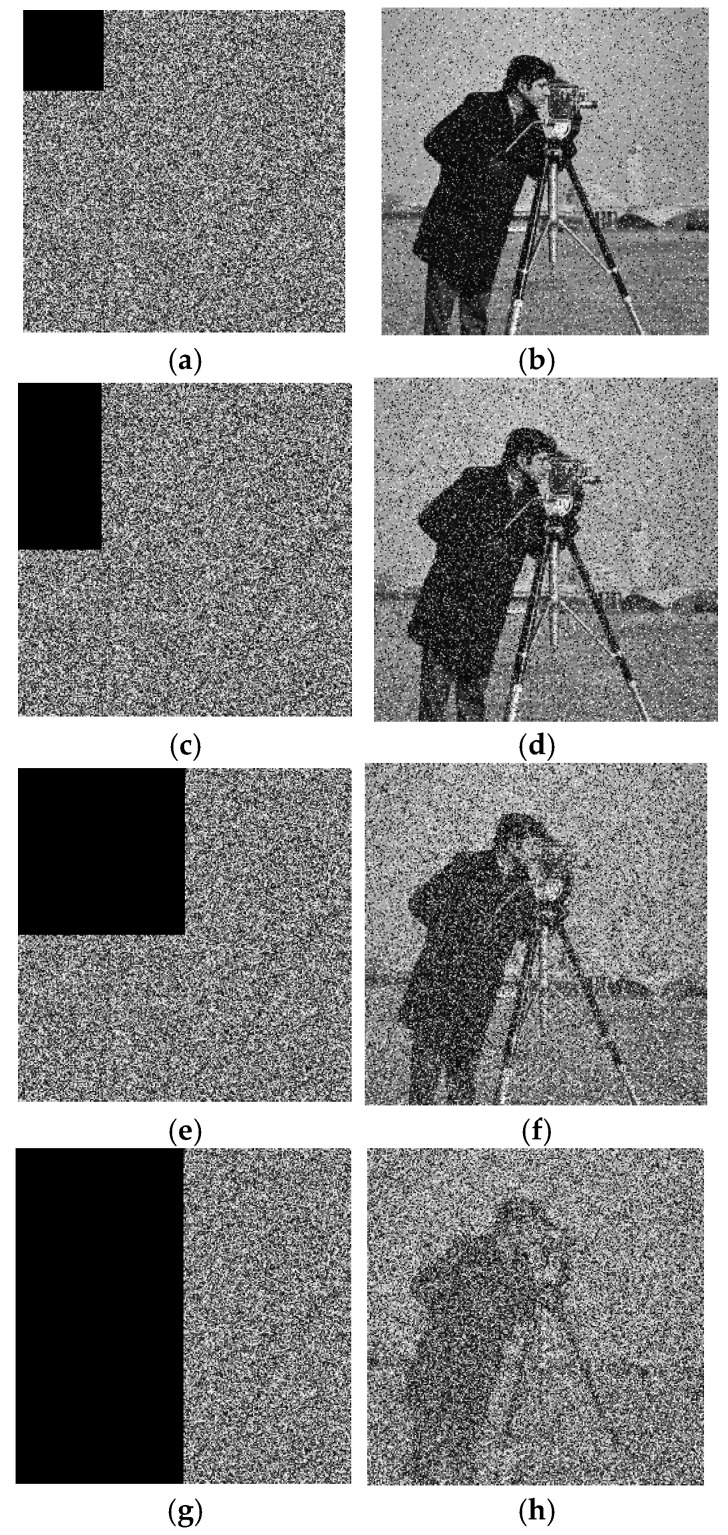
Clipping attack experiment. (**a**) Clipping 1/16 ciphertext image, (**b**) clipping 1/16 decrypted image, (**c**) clipping 1/8 ciphertext image, (**d**) clipping 1/8 decrypted image, (**e**) clipping 1/4 ciphertext image, (**f**) clipping 1/4 decrypted image, (**g**) clipping 1/2 ciphertext image, and (**h**) clipping 1/2 decrypted image.

Further, to test the anti-clipping attack ability of the proposed algorithm, this study set the pixel values of 1/16, 1/8, 1/4, and 1/2 in the ciphertext image to zero and performed decryption. The experimental results are shown in Figure 10, where it can be seen that with the increase in the clipping part, the noise of the decrypted image also increased. Even when the ciphertext image was cut off by half, there was still some information in the decrypted image that could be recognized, indicating that the proposed algorithm had good performance against clipping attacks.

### 7.9. Analysis of Computational Time Complexity

Computational time complexity is an important indicator for evaluating the efficiency of image encryption algorithms. For an image of size M*N, in the encryption algorithm using 4-bit DNA encoding to construct the DNAS_box, due to the parallel nature of bitwise operations in computers, compared to the 2-bit DNA encoding algorithm, the operation speed is the same, and the time complexity for each encoding or decoding is still O(M×N). Compared to traditional DNA encoding encryption algorithms, without considering the shuffling step, this algorithm adds two steps of 4-bit DNA encoding and 4-bit DNA encoding XOR operations, resulting in an overall increase of O(2M×N) in time complexity. In this paper, the time complexity is mainly determined by the generation of chaotic sequences, array Scrambling, and DNA operations, with an overall time complexity of O(21M×N). Table 11 provides a comparison of the time complexities of four DNA encryption algorithms. The algorithm in this paper is at the same order of magnitude as those in references [6,37]. In comparison with conventional DNA-encoded encryption methods, the DNAS_box algorithm presented in this study improves security at the cost of computational efficiency, while preserving linear complexity that remains within practical tolerances.

## 8. Conclusions

This paper proposes a dynamic image encryption algorithm based on the DNA-box and hyperchaotic system. The main features of this algorithm include the use of 2-bit DNA encoding and 4-bit class DNA encoding to construct a DNA-box, achieving the conversion between 2-bit DNA encoding and 4-bit class DNA encoding. This not only separates the encoding and decoding rules but also increases the number of encoding and decoding rules, overcoming the weakness that DNA encoding has only eight effective rules. The algorithm works as follows: first, generate a 256-bit SHA-256 hash value from the original image and perform operations with an external 256-bit key stream to obtain the iterative parameters and initial values of the chaotic system; then, generate DNA and class DNA key streams using the chaotic sequence and dynamically control the encoding rules and operations of the image and DNA keys using the chaotic sequence; subsequently, generate the DNA-box under the control of a mixed sequence, converting DNA encoding to class DNA encoding and performing class DNA encoding operations and decoding under the mixed sequence control; finally, convert the DNA-box into a decimal image matrix to obtain the ciphertext image. Through MATLAB numerical simulations, the algorithm’s security performance, including key space, sensitivity to keys, sensitivity to plaintext, entropy, robustness, and correlation between adjacent pixels, was analyzed, demonstrating strong security performance. However, compared to traditional DNA encoding algorithms, the time complexity of this algorithm has increased slightly but remains within the linear range.

## Figures and Tables

**Table 1:** Eight kinds of DNA encoding and decoding rules.

	Rule1	Rule2	Rule3	Rule4	Rule5	Rule6	Rule7	Rule8
00	A	A	T	T	C	C	G	G
01	C	G	C	G	A	T	A	T
10	G	C	G	C	T	A	T	A
11	T	T	A	A	G	G	C	C

**Table 2:** XOR operations of DNA.

XOR	A	C	G	T
A	A	C	G	T
C	C	A	T	G
G	G	T	A	C
T	T	G	C	A

**Table 3:** Eight kinds of DNA-like encoding and decoding rules.

	Rule1	Rule2	Rule3	Rule4	Rule5	Rule6	Rule7	Rule8
0000	A	M	E	C	D	H	G	I
0001	C	K	C	N	E	F	M	K
0010	E	G	H	I	B	P	A	C
0011	G	O	I	G	K	B	E	M
0100	I	E	A	E	O	L	C	F
0101	K	A	K	P	G	I	J	H
0110	M	I	M	A	J	M	O	P
0111	O	C	O	K	M	C	K	A
1000	P	D	P	L	N	D	L	B
1001	N	J	N	B	I	N	P	O
1010	L	B	L	O	H	J	I	G
1011	J	F	B	F	P	K	D	E
1100	H	P	J	H	L	A	F	N
1101	F	H	G	J	A	O	B	D
1110	D	L	D	M	F	E	N	L
1111	B	N	F	D	C	G	H	J

**Table 4:** XOR operations of DNA-like.

	A	B	C	D	E	F	G	H	I	J	K	L	M	N	O	P
A	A	B	C	D	E	F	G	H	I	J	K	L	M	N	O	P
B	B	A	D	C	F	E	H	G	J	I	L	K	N	M	P	O
C	C	D	A	B	G	H	E	F	K	L	I	J	O	P	M	N
D	D	C	B	A	H	G	F	E	L	K	J	I	P	O	N	M
E	E	F	G	H	A	B	C	D	M	N	O	P	I	J	K	L
F	F	E	H	G	B	A	D	C	N	M	P	O	J	I	L	K
G	G	H	E	F	C	D	A	B	O	P	M	N	K	L	I	J
H	H	G	F	E	D	C	B	A	P	O	N	M	L	K	J	I
I	I	J	K	L	M	N	O	P	A	B	C	D	E	F	G	H
J	J	I	L	K	N	M	P	O	B	A	D	C	F	E	H	G
K	K	L	I	J	O	P	M	N	C	D	A	B	G	H	E	F
L	L	K	J	I	P	O	N	M	D	C	B	A	H	G	F	E
M	M	N	O	P	I	J	K	L	E	F	G	H	A	B	C	D
N	N	M	P	O	J	I	L	K	F	E	H	G	B	A	D	C
O	O	P	M	N	K	L	I	J	G	H	E	F	C	D	A	B
P	P	O	N	M	L	K	J	I	H	G	F	E	D	C	B	A

**Table 5:** An example of a dynamic DNAS_box.

	A	C	G	T
A	L	H	C	F
C	A	K	D	I
G	P	J	O	M
T	E	N	G	B

**Table 6:** The NPCR (%) and UACI (%) when the external key increases $1\times 10^{-14}$.

Index	Variable *x*	Variable *y*	Variable *z*	Variable *w*	Parameter *r*
NPCR	99.63	99.61	99.58	99.55	99.65
UACI	33.65	33.56	33.39	33.48	33.47

**Table 7:** Comparison of information entropy.

Image	Plaintext	Ciphertext	Ref. [6]	Ref. [7]	Ref. [27]	Ref. [35]	Ref. [36]
Cameraman	7.1048	7.9972	7.9970	7.9971	7.9966	7.9955	7.9921
Peppers	7.6013	7.9971	-	7.9971	7.9973	7.9965	7.9934
House	6.4971	7.9970	-	7.9974	7.9989	7.9978	-
All-black	0	7.9975	7.9972	-	-	-	-
All-white	0	7.9976	7.9973	-	-	-	-

**Table 8:** Analysis of the correlation coefficient of adjacent pixels.

Image	Direction	Plaintext	Ciphertext	Ref. [6]	Ref. [7]	Ref. [27]	Ref. [35]
Cameraman	horizontal	0.9593	0.0078	0.0026387	5.0846 × 10^−4^	−0.0211	0.0063
	vertical	0.9357	0.0036	0.010641	0.0020	−0.0103	−0.0142
	diagonal	0.9126	0.0014	−0.000148	1.8576 × 10^−4^	0.0054	0.0168
Peppers	horizontal	0.9540	−0.0036	-	0.0070	−0.0089	0.0194
	vertical	0.9431	0.0060	-	−8.4085 × 10^−4^	−0.0113	−0.0091
	diagonal	0.9138	−0.0052	-	−0.0034	0.0045	0.0123
House	horizontal	0.9632	−0.0027	-	−0.0051	−0.0126	0.0023
	vertical	0.9786	−0.0047	-	0.0022	−0.0097	−0.0187
	diagonal	0.9468	−0.0037	-	0.0055	−0.0123	−0.0225
All-black	horizontal	-	0.0033	-0.0036	-	-	-
	vertical	-	0.0017	0.0261	-	-	-
	diagonal	-	0.0010	0.0033	-	-	-
All-white	horizontal	-	0.0046	-0.0042	-	-	-
	vertical	-	0.0016	0.0187	-	-	-
	diagonal	-	0.0032	-0.0021	-	-	-

**Table 9:** Comparison of NPCR (%) and UACI (%) of differential attack.

Image	Index	Average	Maximum	Minimum	Ref. [6]	Ref. [7]	Ref. [27]	Ref. [37] Average	Ref. [35]
Cameraman	NPCR	99.6078	99.6445	99.5758	99.6002	99.6384	99.6292	99.6067	99.5749
	UACI	33.5237	33.6861	33.3992	33.3921	33.4239	33.7050	33.4634	33.3691
Peppers	NPCR	99.6096	99.6582	99.5606	-	99.6262	99.6092	-	99.5808
	UACI	33.4310	33.5774	33.2945	-	33.4768	33.6284	-	33.3540
House	NPCR	99.6016	99.6552	99.5575	-	99.6246	99.6257	-	99.5332
	UACI	33.4418	33.6177	33.2654	-	33.5114	33.7022	-	33.3912
All-black	NPCR	99.6114	99.6613	99.5651	-	-	-	-	-
	UACI	33.5376	33.6541	33.4471	-	-	-	-	-
All-white	NPCR	99.6092	99.6735	99.5483	-	-	-	-	-
	UACI	33.5369	33.7497	33.3910	-	-	-	-	-

**Table 10:** Cameraman noise experiment.

Ciphertext Image with Noise	Noise Intensity	Decrypted Image	PSNR
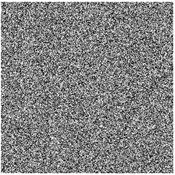	0.005	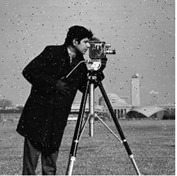	28.0452
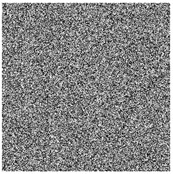	0.01	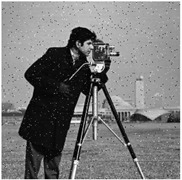	24.8546
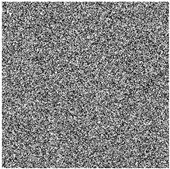	0.05	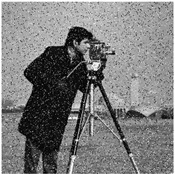	17.8969
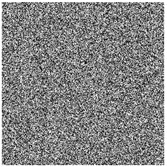	0.1	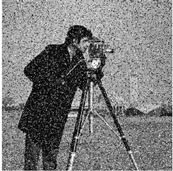	15.0925
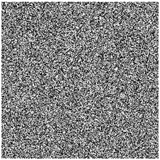	0.2	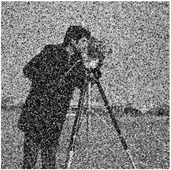	12.5765
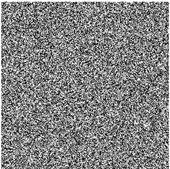	0.3	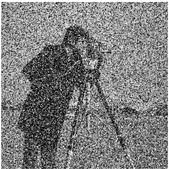	11.1662

**Table 11:** Comparison of computational time complexity.

	Ours	Ref. [6]	Ref. [27]	Ref. [37]	Ref. [35]
Time complexity	O(21MN)	O(14MN)	O(MNlogMN)	$O(11.5N^{2})$	$O(N^{3})$

## Data Availability

The raw data supporting the conclusions of this article will be made available by the authors on request.
